# Alpha and beta spillover in liquid scintillation counting analysis of urine samples

**DOI:** 10.1007/s10967-023-09035-8

**Published:** 2023-07-12

**Authors:** Olga Piraner, Karlee Eardley, Jonathan Button

**Affiliations:** 1 Centers for Disease Control and Prevention, National Center for Environmental Health, Division of Laboratory Sciences, Inorganic and Radiation Analytical Toxicology Branch, 4770 Buford Hwy, MS S110-5, Atlanta, GA 30341-3717, USA; 2 Battelle Memorial Institute, 505 King Avenue, Columbus, OH 43201, USA

**Keywords:** Liquid scintillation counting, Gross alpha/beta urine bioassay, Spillover, Emergency response

## Abstract

Rapid detection and quantification of gross alpha/beta-emitting radionuclides by liquid scintillation counting (LSC) is vital in guiding response to a nuclear or radiological incidents. Liquid scintillation counters use signal pulse shape to discriminate alpha and beta events in samples but require precise optimization to minimize the spillover, or misclassification, of those events. In this study, samples at varying activity levels were analyzed by LSC to determine the effect of activity level, emitter type, and sample matrix on spillover. Analysis proved a matrix effect and a direct correlation of activity level on spillover percentage for both alpha and beta emitting-nuclides.

## Introduction

As part of Centers for Disease Control and Prevention (CDC) radiological/nuclear incident mission preparedness initiative, we are developing methods that will be used to rapidly detect and quantify gross alpha and beta emitting radionuclides in urine samples from populations suspected of internal contamination. Valuable information can be efficiently obtained using Liquid Scintillation Counting (LSC) techniques.

LSC techniques have been continuously developed and used since first appearing in the 1950s [1–3]. The different pulse shape and slower pulse decay times of alpha particles in LSC distinguish alpha particles from other types of radiation [4]. Modern liquid scintillation counters are equipped with a pulse shape analysis (PSA) or a pulse decay discrimination (PDD) option which allows separating alpha signals from beta signals. Due to the PSA/PDD option, alpha and beta radionuclides can be analyzed simultaneously keeping the results in different regions. Instrument-specific PSA/PDD settings can be optimized by preparing spillover curves. Generally, the setting that results in the least spillover is chosen.

Each LSC instrument requires its own calibration. Calibration involves PSA/PDD setting optimization, quench curve preparation at an optimal PSA/PDD setting, and a region of interest (ROI) determination. A ROI is the region in which the given nuclide should be counted [5, 6]. All these parameters affect spillover.

LSC analysis became popular due to its speed in providing analytical results and its reliability. Radiochemists use LSC to analyze gross alpha/beta activities in different matrices [7–9]. There are some publications regarding the quenching and PSA/PDD setting effects on the misclassification of alpha and beta events [10, 11]. However, there is no information on how activity level can affect the alpha/beta spillover. In situations where data analysis yields low activity in one region and high activity in the other, it is difficult to establish if the low activity is a valid analytical result or if it is the result of spillover from high activity.

This work focuses on the relationship between sample activity and spillover in urine through analysis on different LSC instrument models while also investigating sample matrix and emission particle effects.

## Experimental

### Reagents and materials

For gross alpha/beta analysis, we used Ultima Gold^™^ AB cocktail (UGAB) from PerkinElmer and 99% nitromethane from ACROS Organics. Deionized (DI) water was used for all solutions (≥ 18 MΩ∙cm, from an Aqua Solutions Ultrapure Water System, Aqua Solutions, Inc.). All reference solutions used for spiking were purchased from Eckert & Ziegler Analytics, Inc. “Base urine” was collected through anonymous human donations in accordance with the Centers for Disease Control and Prevention Institutional Review Board protocol 3994. We used the base urine to prepare eleven urine samples spiked with Am-241 in the activity range of 15–30,000 Bq/L and eleven separate urine samples spiked with Sr-90/Y-90 in the activity range of 100–300,000 Bq/L. Urine samples spiked with different activities of Co-60, Cs-137, and Am-241 (all three nuclides together) were prepared by Eckert & Ziegler Analytics as well as urine gross alpha/beta quality control (QC) materials (GAB-QC-Low and GAB-QC-High). The QC materials were spiked into base urine that was acidified to 1% nitric acid to yield two quality control pools with Am-241 and Sr-90/Y-90 at high and low levels. QC materials were stored in a freezer at approximately − 70°C. All radioactive source solutions were traceable to the National Institute for Standards and Technology (NIST) (Gaithersburg, MD, USA).

### Instrumentation and labware

We used six instruments for our LSC analysis. The instruments will be referred to as Quantulus^™^1220, Tri-Carb^®^3110 #1, Tri-Carb^®^3110 #2, Tri-Carb^®^5110, Quantulus^™^GCT6220 #1, and Quantulus^™^GCT6220 #2 (all PerkinElmer). We used 20 mL LSC plastic vials (PerkinElmer); a high-precision analytical balance with an accuracy of 0.0001 g (Mettler-Toledo, LLC); 50 mL conical poly-propylene tubes (Becton Dickinson) for solution preparation; a Brinkman bottle top dispenser with 5 mL to 25 mL capacity (Brinkman Instruments, Inc.) for cocktail dispensing; and four electronic pipettes with a total volume range from 5 μL to 5 mL (Eppendorf, Inc).

### Sample preparation and LSC analysis

LSC instrument optimization was performed as described in our previous publications [5, 6]. An optimal PSA or PDD setting was determined for each instrument. Quench curves were prepared at chosen PSA/PDD settings according to procedures for Am-241 (alpha nuclide) and Sr-90/Y-90 (beta nuclide). We used 20 mL of UGAB and nitromethane as a quenching agent. We optimized sample analysis time, external standard analysis time, sample/cocktail volume for 20 mL vial geometry, and the region of interest (ROI) for each instrument. These parameters are summarized in Table 1. Finally, an aliquot (5 mL) of each urine sample was mixed with 15 mL of UGAB cocktail in a 20-mL LSC plastic vial until it was homogenous. The LSC vials were placed in the LSC counter and LSC analysis was performed.

## Results and discussion

**Fig.1:**
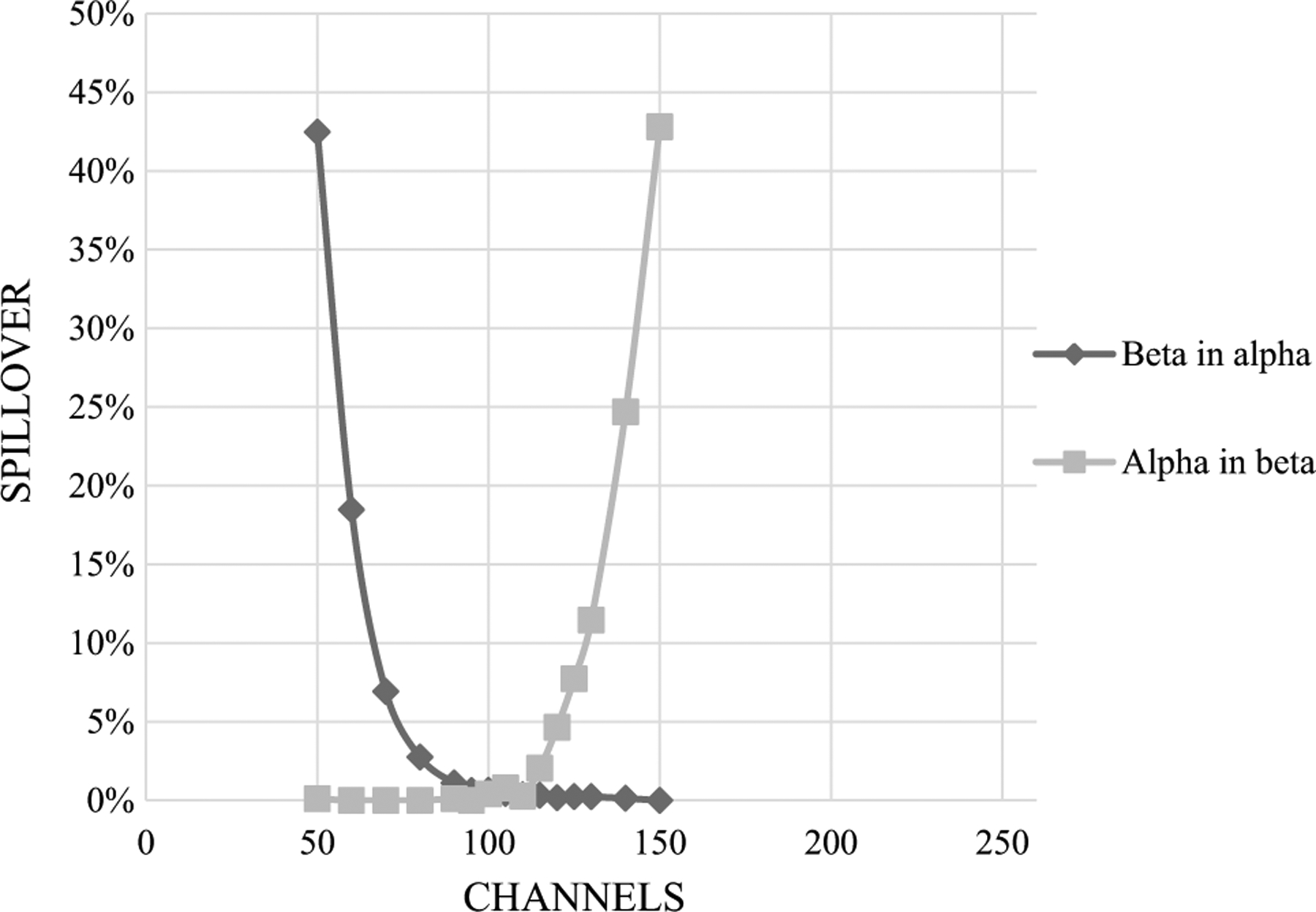
Am-241 and Sr-90/Y-90 Spillover curves on Quantulus1220 in DI water x-axis is for PSA setting, optimal PSA = 100, spillover–0.2%

**Fig.2:**
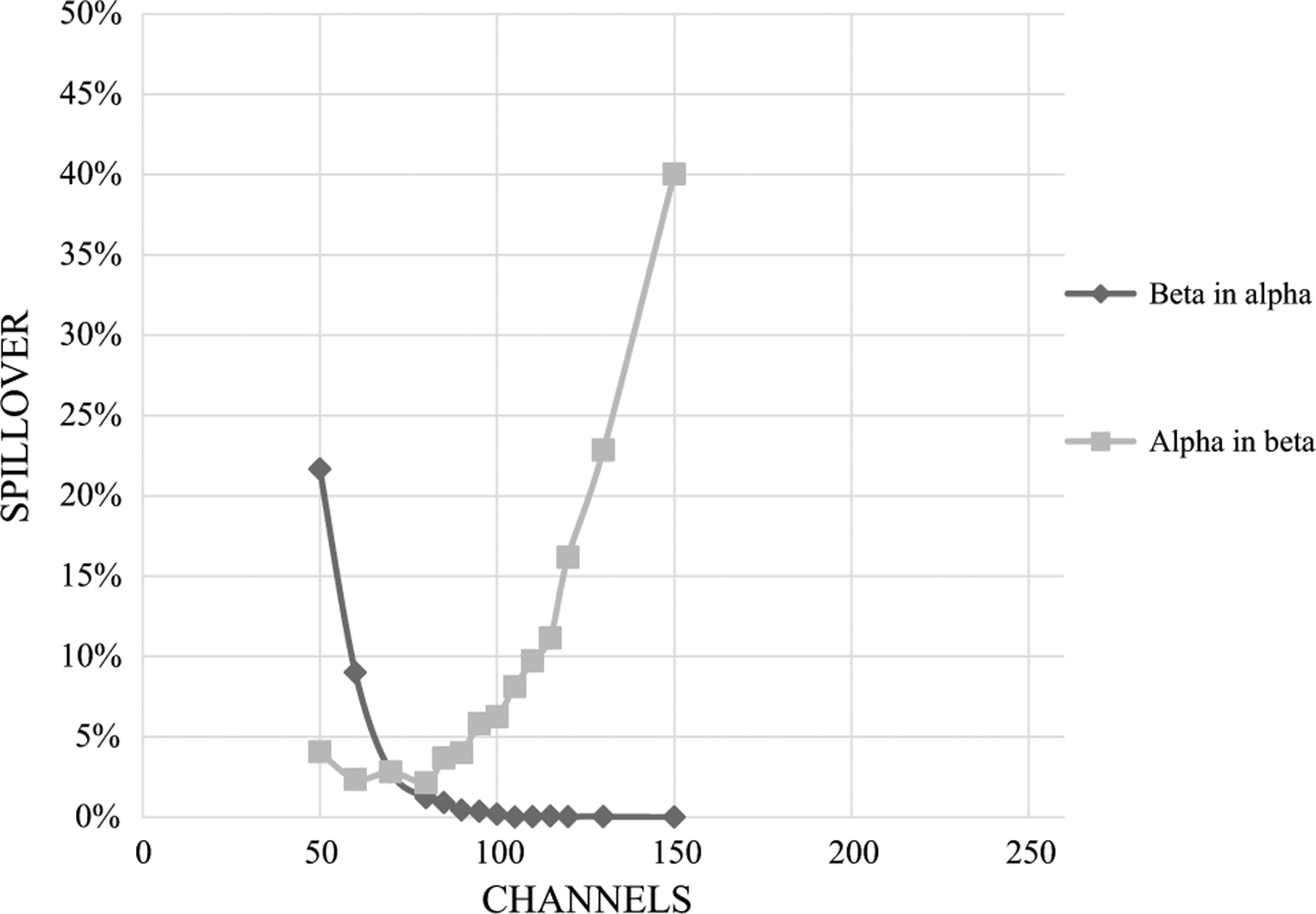
Am-241 and Sr-90/Y-90 Spillover curves on Quantulus1220 in base urine x-axis is for PSA setting, optimal PSA = 80, spillover–3.4%

**Fig.3:**
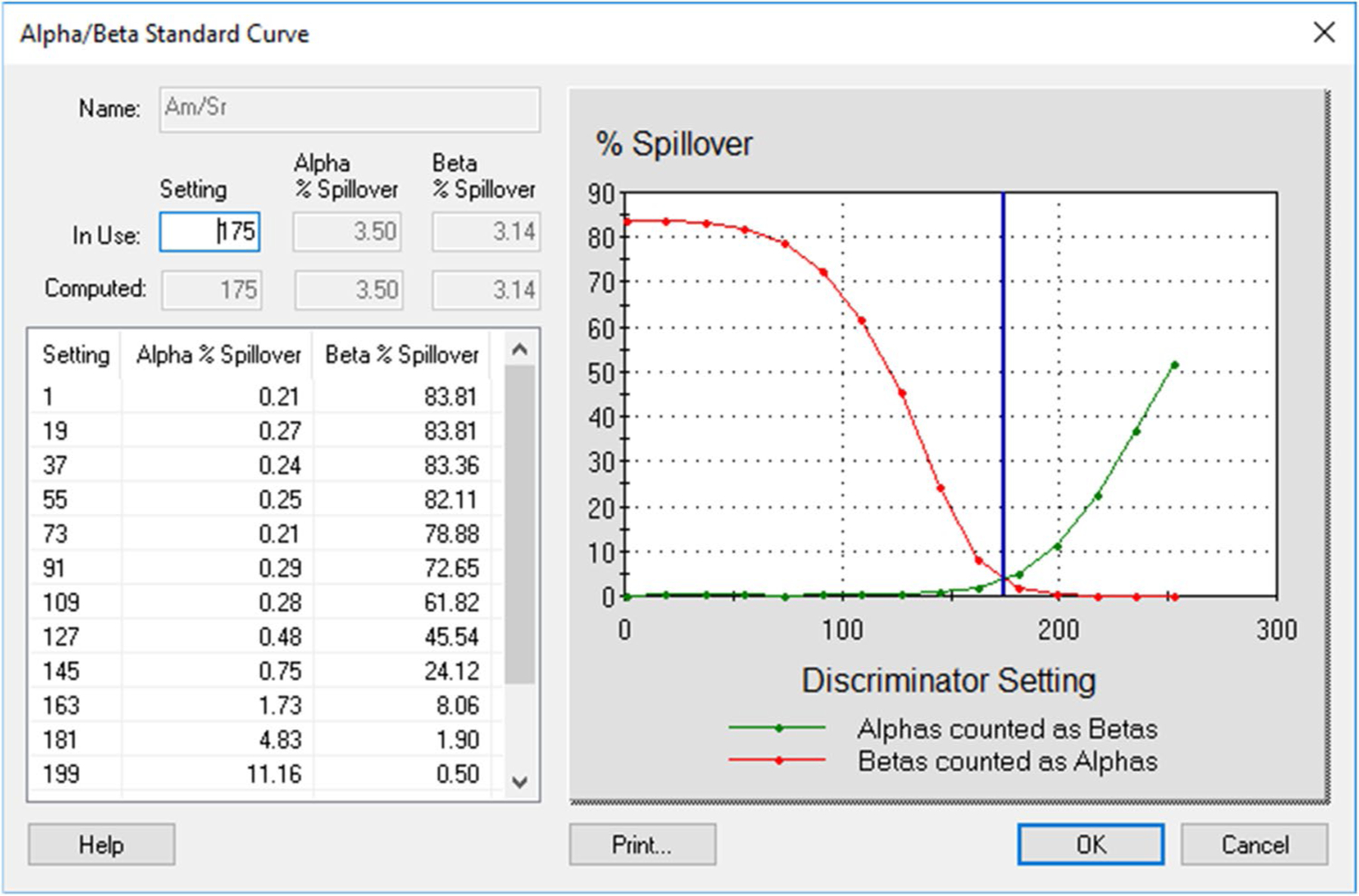
Am-241 and Sr-90/Y-90 Spillover curves on Tri-Carb3110#2 in base urine for high activities (Am-241 at 30,000 Bq/L and Sr-90/Y-90 at 300,000 Bq/L), x-axis is in keV

**Fig.4:**
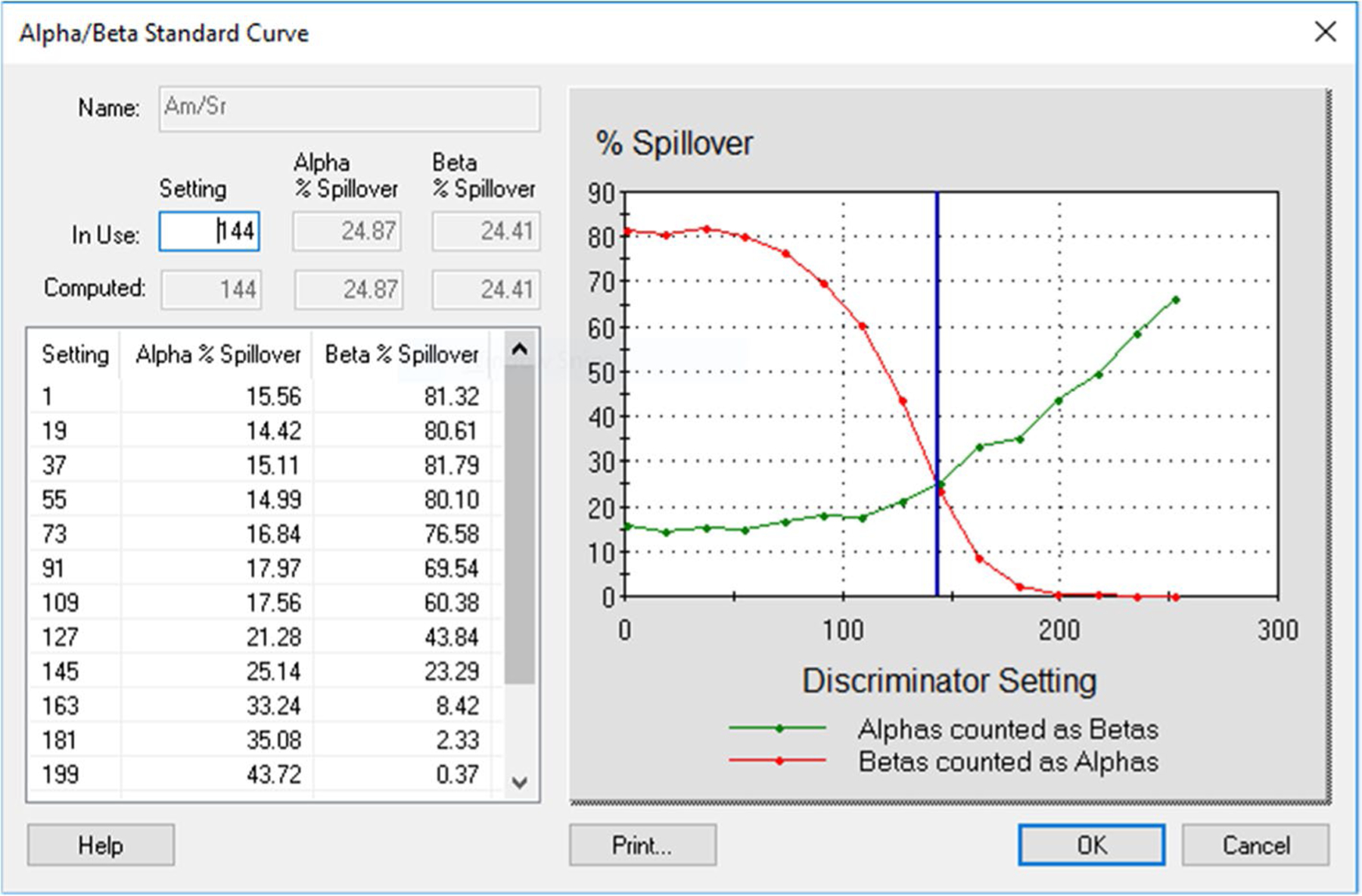
Am-241 and Sr-90/Y-90 Spillover curves on Tri-Carb3110#2 in base urine for low activities (Am-241 at 300 Bq/L and Sr-90/Y-90 at 3000 Bq/L), x-axis is in keV

**Fig.5:**
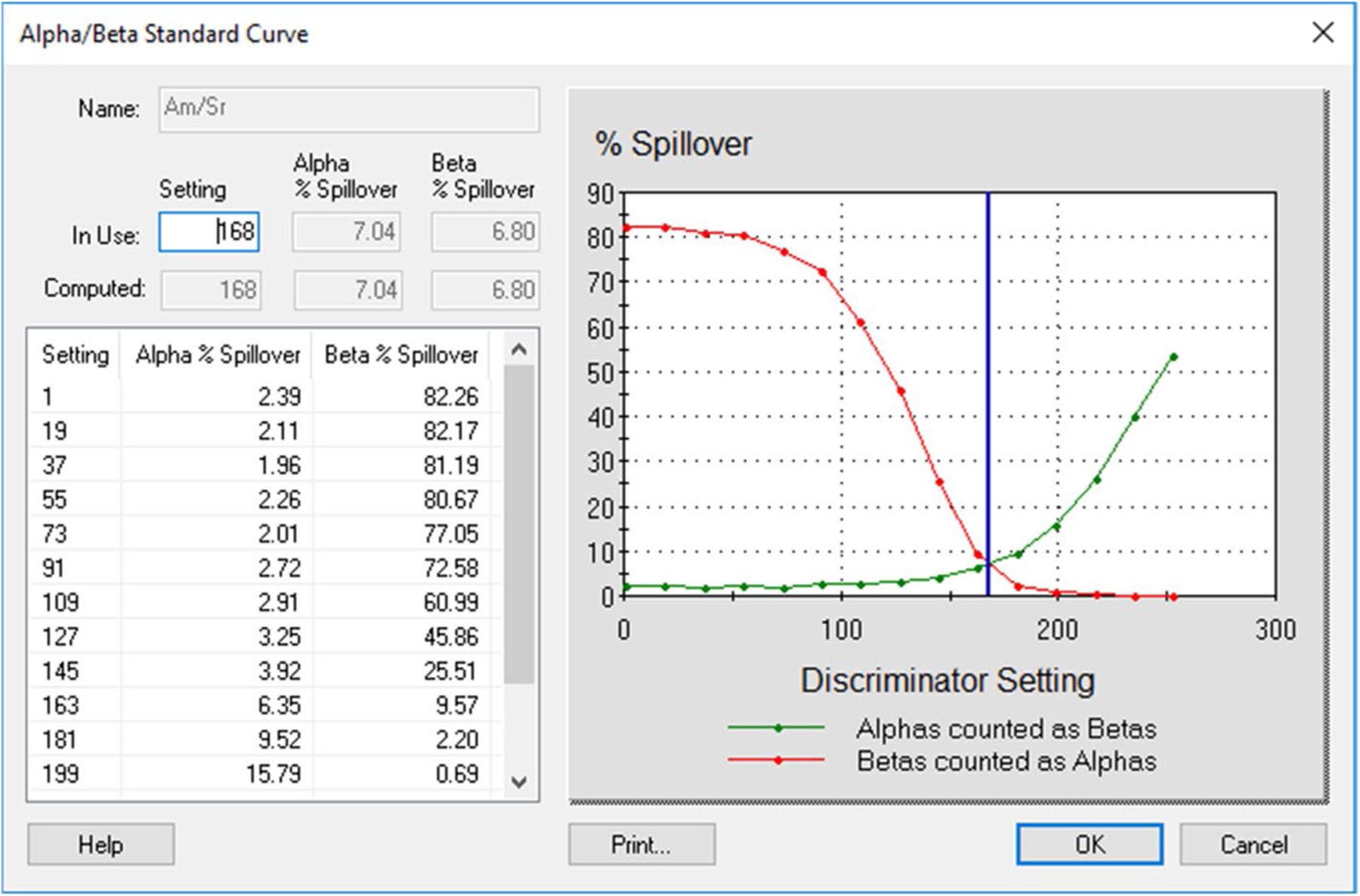
Am-241 and Sr-90/Y-90 Spillover curves on Tri-Carb3110#2 in base urine for medium activities (Am-241 at 3000 Bq/L and Sr-90/Y-90 at 3000 Bq/L), x-axis is in keV

First, we investigated the effect of matrices on spillover between alpha and beta radionuclides by preparing spillover curves using DI water and separate spillover curves using base urine. It was found that the matrix (urine vs DI water) affected the spillover. The PSA/PDD setting was higher, and spillover was smaller in DI water than in urine (Figs. 1 and 2). These graphs, based on Quantulus 1220, were presented as examples. Other instruments gave similar results. An optimal PSA/PDD setting was chosen between the settings for DI water and base urine. We also studied the effect of activity level of alpha and beta nuclides used for PSA/PDD setting optimization. We found that nuclides with lower activities had lower PSA/PDD settings and higher spillover than nuclides with higher activities (see Fig. 3, 4 and 5 as examples, based on Tri-Carb3110 #2). Therefore, the amount of activity used for spillover curves preparation should be taken into consideration for PSA/PDD setting optimization. For the automatic spillover curves preparation method, the nuclides activities should be no less than 50,000 counts per minute (CPM) or about 830 Bq per sample. When possible, we used a manual spillover curve preparation method and nuclides activity in the range around 100 Bq/sample.

Next, the region of interest (ROI) was chosen to minimize spillover on each instrument. The results of this optimization were presented in Table 1 together with other method parameters [5, 6] and the limit of detection (LOD) found for each instrument [12].

Finally, we investigated the effect of radionuclide activity on spillover. For this purpose, two sets of urine spikes were prepared: Am-241 urine set with activity in the range of 15–30000 Bq/L and Sr-90/Y-90 with activity in the range of 100–3,000,000 Bq/L. We analyzed these spiked urine samples at the optimal parameters on each instrument 3 times on different days, once per day. We used average activity results that are presented in Tables 2 and 3. Urine low and high gross alpha/beta QC materials were analyzed with each set of samples at the beginning and end of the run.

The data showed that Quantulus1220 does not show alpha spillover in the beta region until alpha activity reaches 10,000 Bq/L. This probably can be explained by the different instrument design. Quantulus 1220 has the biggest lead shielding in comparison with other LSC instruments, which provides better defense from the cosmic radiation. Therefore, it has the lower background and better separation between alpha and beta particles. Beta signal found during analysis of the urine samples spiked with Am-241 (less than 10,000 Bq/L) belongs to the base urine used for spike preparation. The base urine always contains some beta nuclides, predominantly from the small percentage of dietary potassium that is radioactive (K-40) [13]. The Tri-Carb and GCT series LSC instruments showed increased beta signals with Am-241 urine spike of 5000 Bq/L.

During the spillover analysis of urine samples spiked with Sr-90/Y-90, we found that Quantulus 1220 did not show any spillover until Sr-90/Y-90 activity reached 1000 Bq/L. After that, the beta spillover in the alpha region was obvious and continues to grow with growing beta activity. The spillover reached 2100 Bq/L for 300,000 Bq/L of gross beta activity—about 0.7% of the total beta activity. The GCT6220 series LSC instruments showed small beta spillover into the alpha region that was less than the LOD for a total beta activity of 300 Bq/L. Tri-Carb series LSC instruments showed spillover of about 8–14 Bq/L for the same urine spikes. For the Sr-90/Y-90 urine sample spiked with 300,000 Bq/L activity, the spillover for the GCT6220 instruments series was 4500–5700 Bq/L or about 1.7% of total beta activity. For the Tri-Carb series instruments, the spillover for the 300,000 Bq/L Sr-90/Y-90 urine spike was 7500–8300 Bq/L or about 2.6% of total beta activity.

The LSC screening of 100 urine samples (Eckert & Ziegler Analytics) showed that 20 samples contained some alpha and beta nuclides. The activity of beta nuclides was in the range of 3400–33,800 Bq/L. The activity of alpha nuclides in the range of 34–870 Bq/L was dependent on beta activity. The higher beta activity samples showed higher alpha activity. We examined whether the alpha signals in these samples were an indicator for real alpha activity or high activity beta spillover into the alpha region. The measurement results and the target data are presented in Table 4. As indicated, the urine samples were spiked with some amount of beta emitters: Co-60 and Cs-137 in the range of 3400 – 30,000 Bq/L and some amount of alpha: Am-241 in the range of 0.1–0.5 Bq/L. The amount of Am-241 was too low to see by LSC. The Am-241 amount is much less than the LOD of any LSC instrument used for analysis (Table 1). Comparing the real target values, we realized that the observed alpha signals were due to high activity beta spillover into the alpha region. The fact that these alpha signals do not correlate with the spiked amount of alpha activity, but they do only depend on beta activities also indicates a beta signal spillover effect. Am-241 was analyzed by another method which required purification and sector field mass-spectrometry [14].

The measured gross beta activities were slightly higher than target for most of the samples. This accuracy bias was due to the use of the Sr-90 quench curve to calculate gross beta activity from sample measurements. The LSC Sr-90 efficiency is generally 10% lower than the LSC efficiency for Co-60 or Cs-137, and this discrepancy in efficiency accounts for the observed positive bias.

## Conclusion

This study confirmed that alpha/beta spillover takes place during LSC analysis. PSA/PDD and ROI optimization help to decrease but not eliminate the spillover. As expected, the radionuclide activity affects the spillover: the higher the activity the higher the spillover. More importantly, the spillover also depends on the LSC instrument. For LSC Tri-Carb and GCT6220 series instruments, Am-241 started showing spillover in the beta region at an activity of 5000 Bq/L. In contrast, Sr-90/Y-90 produced spillover in the alpha region starting at 300 Bq/L. The most lead-shielded instrument (Quantulus 1220) started showing spillover at higher activities for both alpha and beta emitters. The instruments in the GCT6220 series, which are Tri-Carbs equipped with guard compensation technology (GCT), showed smaller spillover than the Tri-Carb series instruments for the same activity samples.

It should be noted that when a sufficient level of both alpha and beta activity was present in a sample, spillover effect was seemingly negligible. Data for quality control materials containing both Am-241 and Sr-90/Y-90 nuclides, presented in Tables 2 and 3, support this conclusion. However, when urine contains one nuclide at much higher activity than another, the results for the lower activity nuclide may be questionable because of spillover. The urine spikes from Eckert & Ziegler Analytic demonstrated this very well. None of the LSC instruments used can analyze Am-241 at such low levels in 5 min; therefore, the observed signal in the alpha area was just beta spillover. In such cases, more specific and precise methods are needed for radionuclides analysis.

## Figures and Tables

**Table 1:** LSC method parameters for different instruments [5, 6, 12]

Parameter	LSC Instruments (PerkinElmer)					
	Tri-Carb3110 #1	Tri-Carb3110 #2	Tri-Carb5110	Quantulus GCT6220 #1	Quantulus GCT6220#2	Quantulus 1220
PSA/PDD (keV)	125	165	135	160	160	90
Sample volume	5 mL	5 mL	5 mL	5 mL	5 mL	5 mL
Cocktail volume	15 mL	15 mL	15 mL	15 mL	15 mL	15 mL
Sample analysis time	5 min	5 min	5 min	5 min	5 min	5 min
External Std analysis time	2 Ω (15 s)	2 Ω (15 s)	2 Ω (10 s)	60 s	60 s	60 s
Alpha ROI	0–300 keV	0–200 keV	0–1000 keV	0–450 keV	0–1000 keV	1–1024 Ch
High energy Beta ROI	0–2000 keV	0–2000 keV	0–2000 keV	0–2000 keV	0–2000 keV	1–1024 Channel
LOD for gross alpha, Bq/L	12.6	12.6	12.6	7.6	7.6	5.8
LOD for gross beta, Bq/L	44.6	44.6	44.6	40.3	40.3	31.3

**Table 2:** LSC gross alpha/beta analysis of Am-241 urine spikes with different activities

Sample ID	Analyte	Gross alpha/beta specific activity by instrument, Bq/L						Target (SD) Bq/L
		Q1220	TC3110#1	TC3110 #2	TC5110	GCT#1	GCT#2	
GAB-Low-QC Average	gAlpha	85	91	80	72	82	93	85 (9)
	gBeta	1760	1756	1740	1818	1765	1737	1740 (48)
GAB-High-QC Average	gAlpha	5260	5142	5190	5218	5933	5914	5370 (438)
	gBeta	104,700	105,000	104,900	106,100	105,160	104,500	105,000 (1650)
Base urine	gAlpha	0.7	1.5	1.2	1.6	0.0	0.9	
	gBeta	60	49	55	52	55	48	
Am241-Spike1	gAlpha	14	15	11	11	15	13	14 (3)
	gBeta	55	60	72	41	50	57	
Am241-Spike2	gAlpha	49	48	48	43	50	48	50 (7)
	gBeta	59	43	60	54	52	52	
Am241-Spike3	gAlpha	95	105	97	93	92	101	100 (9)
	gBeta	64	47	58	59	51	43	
Am241-Spike4	gAlpha	497	493	468	482	456	477	500 (27)
	gBeta	60	42	75	54	58	47	
Am241-Spike5	gAlpha	1006	1018	995	956	952	976	1000 (41)
	gBeta	50	62	72	56	60	59	
Am241-Spike6	gAlpha	1994	1962	1960	1924	1933	1961	2000 (88)
	gBeta	55	75	76	51	65	61	
Am241-Spike7	gAlpha	5234	5169	5028	5007	4955	5110	5200 (179)
	gBeta	66	102	132	65	87	82	
Am241-Spike8	gAlpha	10,188	9992	10,011	9675	9612	9955	10,200 (371)
	gBeta	72	173	187	60	127	138	
Am241-Spike9	gAlpha	14,625	14,353	14,214	13,983	13,873	14,358	14,700 (430)
	gBeta	78	238	262	72	152	171	
Am241-Spike10	gAlpha	19,902	19,358	19,174	18,855	18,759	18,734	19,800 (513)
	gBeta	81	283	257	75	174	187	
Am241-Spike11	gAlpha	29,206	28,783	28,464	27,890	27,794	28,072	30,000 (660)
	gBeta	89	401	407	86	254	283	

**Table 3.** LSC gross alpha/beta analysis of Sr-90/Y-90 urine spikes with different activities

Sample ID	Analyte	Gross alpha/beta specific activity by instrument, Bq/L						Target (SD)
		Q1220	TC3110#1	TC3110#2	TC5110	GCT#1	GCT#2	Bq/L
	gAlpha	91	79	90	85	83	86	85 (9)
GAB-Low-QC Average	gBeta	1760	1780	1740	1800	1760	1740	1740 (48)
GAB-High-QC Average	gAlpha	5250	5030	5180	5160	5920	5980	5370 (438)
	gBeta	104850	105300	104900	105300	104800	104890	105000 (1650)
Base Urine	gAlpha	0	0.9	2.9	1.6	1.0	0	
	gBeta	54	61	60	49	45	42	
Sr90-Spike1	gAlpha	0.4	8.6	2.4	1.0	0.3	0.0	
	gBeta	114	102	103	104	115	101	110 (11)
Sr90-Spike2	gAlpha	1.6	8.2	9.1	4.9	3.6	2.8	
	gBeta	236	243	240	256	255	242	250 (14)
Sr90-Spike3	gAlpha	1.1	11	8.2	14	3.8	3.6	
	gBeta	343	317	335	331	325	319	350 (18)
Sr90-Spike4	gAlpha	5.5	26	20	28	13	10	
	gBeta	1070	1070	1030	1080	1070	1060	1070 (32)
Sr90-Spike5	gAlpha	15	56	43	48	29	35	
	gBeta	2030	1950	1940	2010	1960	2010	2010 (44)
Sr90-Spike6	gAlpha	62	244	228	261	143	189	
	gBeta	10120	9550	9530	9950	9980	9970	10010 (253)
Sr90-Spike7	gAlpha	142	505	470	516	287	381	
	gBeta	20500	19580	19160	20160	20090	20170	20100 (530)
Sr90-Spike8	gAlpha	273	993	949	1073	580	759	
	gBeta	40600	38700	38100	39800	40000	40000	40030 (955)
Sr90-Spike9	gAlpha	654	2340	2300	2630	1420	1820	
	gBeta	96100	91700	90800	94400	94500	95100	95700 (2080)
Sr90-Spike10	gAlpha	1500	5090	5070	5820	3120	3890	
	gBeta	203000	194000	192000	200000	201000	201000	203000 (4425)
Sr90-Spike11	gAlpha	2120	7530	7520	8260	4520	5670	
	gBeta	296000	282200	280000	292000	292000	292000	296000 (6600)

**Table 4:** The LSC results for Eckert & Ziegler Analytics urine spikes analysis with target data

Sample ID	Found activities with uncertainty (2SD), Bq/L				Instrument ID	Target Activities, Bq/L	
	gBeta	Uncertainty	gAlpha	Uncertainty		Target Co-60 and Cs-137	Target Am-241
57,836	3415	97	71	13	GCT#1	3421	0.5
57,849	5289	119	37	8	TC5110	3942	0.2
57,868	4361	108	43	9	TC5110	3941	0.2
57,892	3816	100	102	16	TC3110#2	3421	0.5
57,920	3804	101	89	15	TC3110#1	3421	0.5
57,931	4256	107	94	16	TC3110#1	3421	0.2
57,842	15,853	209	329	30	GCT#1	15,542	0.1
57,861	17,335	217	172	20	TC5110	15,542	0.1
57,884	16,364	210	446	34	TC3110#2	14,604	0.5
57,899	16,459	211	449	35	TC3110#2	15,542	0.1
57,906	16,654	213	449	34	TC3110#1	14,604	0.5
57,910	16,461	212	433	34	TC3110#1	14,604	0.5
57,914	17,196	217	489	36	TC3110#1	15,542	0.1
57,844	31,517	284	660	42	GCT#1	29,104	0.2
57,858	33,691	303	339	29	TC5110	29,521	0.1
57,862	33,821	304	346	29	TC5110	29,521	0.1
57,871	33,430	301	335	29	TC5110	29,104	0.2
57,875	33,100	298	872	48	TC3110#2	29,521	0.1
57,903	32,715	298	845	47	TC3110#2	29,104	0.2
57,917	33,403	304	869	48	TC3110#1	29,521	0.1
